# A Rapid and Simple Method for Lithium Purification and Isotopic Analysis of Geological Reference Materials by MC-ICP-MS

**DOI:** 10.3389/fchem.2020.557489

**Published:** 2020-11-23

**Authors:** Guanhong Zhu, Jinlong Ma, Gangjian Wei, Le Zhang

**Affiliations:** ^1^State Key Laboratory of Isotope Geochemistry, Guangzhou Institute of Geochemistry, Chinese Academy of Sciences, Guangzhou, China; ^2^University of Chinese Academy of Sciences, Beijing, China

**Keywords:** lithium purification, Li isotopes, MC-ICP-MS, geological reference materials, AGMP-50

## Abstract

A simple method has been developed to purify lithium (Li) from matrix elements in geological reference materials, using a single-column packed with AGMP-50 cation exchange resin, followed by high-precision Li isotope measurements by multi-collector inductively coupled plasma mass spectrometry (MC-ICP-MS). A series of tests, such as different types of resin, loading amount of Li, loading volumes, and various eluents, were conducted to ascertain the optimal conditions for Li purification and the effects of intensity, acidity, and presence of potential matrix elements on Li isotope measurements were also evaluated. In our experiment, Al and high-field-strength elements (HFSEs), such as Ti, Zr, and Hf, were eluted by 0.2 M HCl + 0.3 M HF, and 0.73 M HCl was used to separate Li from other matrix elements, such as Na. This method is suitable for processing large amount of Li (60–270 ng) and enabling a Li recovery of close to 100%, with effective removal of matrix elements such as Na and Ca. Besides, our method achieves low matrix interferences (e.g., Na/Li << 1 and Ca/Li << 1 for rock and seawater via a single-column procedure; Ca/Li < 2 for carbonate via a two-column procedure) and also uses small volume of eluents and is rapid (~5 h), enabling a total separation to be completed in ~0.5 d. Using this method, we report Li isotopic compositions of various geological reference materials, including igneous rocks, seawater, and carbonate. The Li isotopic compositions are consistent with the data published previously for the analyzed reference materials. As such, the reported method is ideally suited for Li separation from multiple types of geological samples prior to isotopic analysis.

## Introduction

Lithium is a moderately incompatible element in magmas and, therefore, is preferentially enriched in the crust and depleted in the mantle during crust–mantle differentiation (Rudnick et al., 2004). Significant Li isotopic fractionations occur during deep geological processes (Chan et al., 1994; Elliott et al., 2006; Li J. et al., 2018) and silicate weathering (Kisakurek et al., 2004; Teng et al., 2010; Misra and Froelich, 2012; Tsai et al., 2014; Lechler et al., 2015; Sun et al., 2018), which make Li isotopes an important proxy for various geochemical processes. Consequently, it is quite essential to conduct high-precision and accurate Li isotopic measurements on geological samples.

Accurate and precise measurements of Li isotopes were initially performed by thermal ionization mass spectrometry (TIMS) (Michiels and De Bièvre, 1983; Chan, 1987; Xiao and Beary, 1989; Moriguti and Nakamura, 1993). However, the TIMS technique suffers from a highly instable instrumental fractionation and also time consuming (Tomascak et al., 1999; Bryant et al., 2003; Jeffcoate et al., 2004; Magna et al., 2004). In comparison, MC-ICP-MS offers several distinct advantages including rapid sample analysis (~8 min) and high precision (~0.5%0 2SD). Therefore, Li isotope measurements are typically conducted by MC-ICP-MS at present. Prior to this, Li purification is generally carried out using one or more columns packed with cation exchange resins, such as AG50W-X8 and/or AG50W-X12 (BioRad), to separate Li from matrix elements using HNO_3_ and methanol eluents (Tomascak et al., 1999; Nishio and Nakai, 2002; Jeffcoate et al., 2004; Magna et al., 2004; Rosner et al., 2007; Huang et al., 2010). The addition of an organic eluent results in a better separation of Li from Na. However, as noted by Li W. et al. (2018), this is time-consuming and generally requires a large volume of eluents, and thus increasing the risk of contamination in Li blanks. Besides, several previous studies (Misra and Froelich, 2009; Gao and Casey, 2012; Van Hoecke et al., 2015; Bastian et al., 2018; Li W. et al., 2018) have used low-concentration HCl as the eluent to purify Li from matrix elements. For example, Gao and Casey (2012) used a two-column procedure with AG50W-X8 and AG50W-X12 (BioRad™) cation exchange resins and 0.2 M HCl (i.e., 0.2 mol/L) as an eluent to purify Li. However, this method requires large elution volume (up to 246 mL). Li W. et al. (2018) also used a two-column procedure with AG50W-X8 resin and 0.2 M and 0.5 M HCl as eluents to purify Li, which also involved a large elution volume (62 mL) and takes ~1.5 d to complete the entire purification procedure. Bohlin et al. (2017) used a single-column procedure with AGMP-50 cation exchange resin and 0.5 M HF and 0.7 M HCl as eluents to purify Li and Mg from other matrix elements. This Li purification procedure takes ~1.0 d and has a relatively low elution volume of 26 mL, but is only suitable for a small amount of Li (from 0.3 to 20 ng). However, this method is not appropriate for Li isotope measurements on a MC-ICP-MS equipped with 10^11^ and 10^12^ Ω resistors. For example, 50 ~ 100 ng of Li is needed for high-precision Li isotopic determinations on a MC-ICP-MS with 10^11^ Ω resistors. In addition, digesting too small amount of samples has potential problems in terms of sample homogeneity and procedural blanks. Therefore, it is desirable to process larger samples and more Li.

The different amount of Li loaded onto a column can lead to a shift in the elemental elution curves. As such, we conducted a series of tests, whereby the Li loading amount, loading volumes, and eluents were varied, in order to ascertain the optimal conditions for Li purification using AGMP-50 cation exchange resin. We also compared the Li separation efficiency of three types of commonly used cation exchange resins, and evaluated the effects of intensity, acidity, and presence of potential matrix elements on Li isotopic measurements by MC-ICP-MS. Finally, we present a new, optimized and simple method for Li purification and high-precision Li isotope measurements by MC-ICP-MS.

## Experimental Methods

### Chemical Reagents

Ultra-pure water (Milli-Q) with a resistivity of 18.2 MΩ·cm was purified with a Millipore system (USA). Hydrofluoric (HF) and hydrochloric (HCl) acids were sub-boiling distilled from BVIII grade acids (electronic grade) with a Savillex DST-1000 system (USA), while nitric acid (HNO_3_) was double-distilled from GR grade acid with the same system. The distilled HCl and HF were diluted with Milli-Q water to appropriate concentrations.

### Sample Digestion

A suite of reference materials were used in this study, including granite (JG-2), basalt (BHVO-2, BCR-2, and JB-2), andesite (JA-2 and AGV-2), and rhyolite (JR-2). To reduce the effects of procedural blanks and sample heterogeneity, 7–70 mg of each rock standard (containing 270 to 540 ng of Li) was weighed into a pre-cleaned 7 mL PFA Savillex® beaker. Firstly, 0.5 mL of 8 M HNO_3_ and 1 mL of concentrated HF were added to the samples, which were then capped tightly and placed on a hot plate at 120°C for 1 h. The solutions were then evaporated to dryness, another 1 mL of 8 M HNO_3_ and 1 mL of concentrated HF were added, and the beakers were placed on a hot plate for 7 d. Following evaporation to dryness, another 1.5 mL of concentrated HNO_3_ was added, the beakers were capped and placed on a hot plate for at least 1 h, and then the solutions were evaporated again. 1.2 mL of aqua regia was then added to re-dissolve the samples, which were heated for 4 h at 120°C. Following this step, the solution was dried again and re-dissolved in 2 mL of 6 M HCl. This solution was dried and finally re-dissolved in 1 mL of 2.5 M HCl for ion exchange chromatography.

The carbonate standard JCp-1 was weighed (0.27 g; 120 ng of Li) into a pre-cleaned 7 mL PFA Savillex® beaker. A total of 1 mL of 2.5 M HCl and 0.5 mL of 6 M HCl was added dropwise until the sample was completely dissolved, which was then placed on a hotplate at 120°C for 6 h. After evaporation to dryness, 1 mL of 6 M HCl was added to re-dissolve the sample, which was capped and heated for 4 h at 120°C. Finally, the solution was dried again and re-dissolved in 1 mL of 2.5 M HCl for ion exchange chromatography.

The seawater standard IAPSO (600 μL; 120 ng of Li) was evaporated to dryness and re-dissolved in 1 mL of 2.5 M HCl for ion exchange chromatography.

### Ion Exchange Chromatography

Previous studies have shown that when the total ionic load is <10% of the total resin capacity in terms of milliequivalents (meq = mg × valence/molar mass), the ion selectivity of the resin is maintained (James and Palmer, 2000; Gao and Casey, 2012). The capacity of AGMP-50 (200–400 mesh) resin is 4.6 meq/g (dry resin). As such, 1.4 g of AGMP-50 resin (200–400 mesh; total capacity = 6.44 meq) was packed into polypropylene columns, with diameters of ~0.7 cm and lengths of ~8.5 cm.

**Figure 1:**
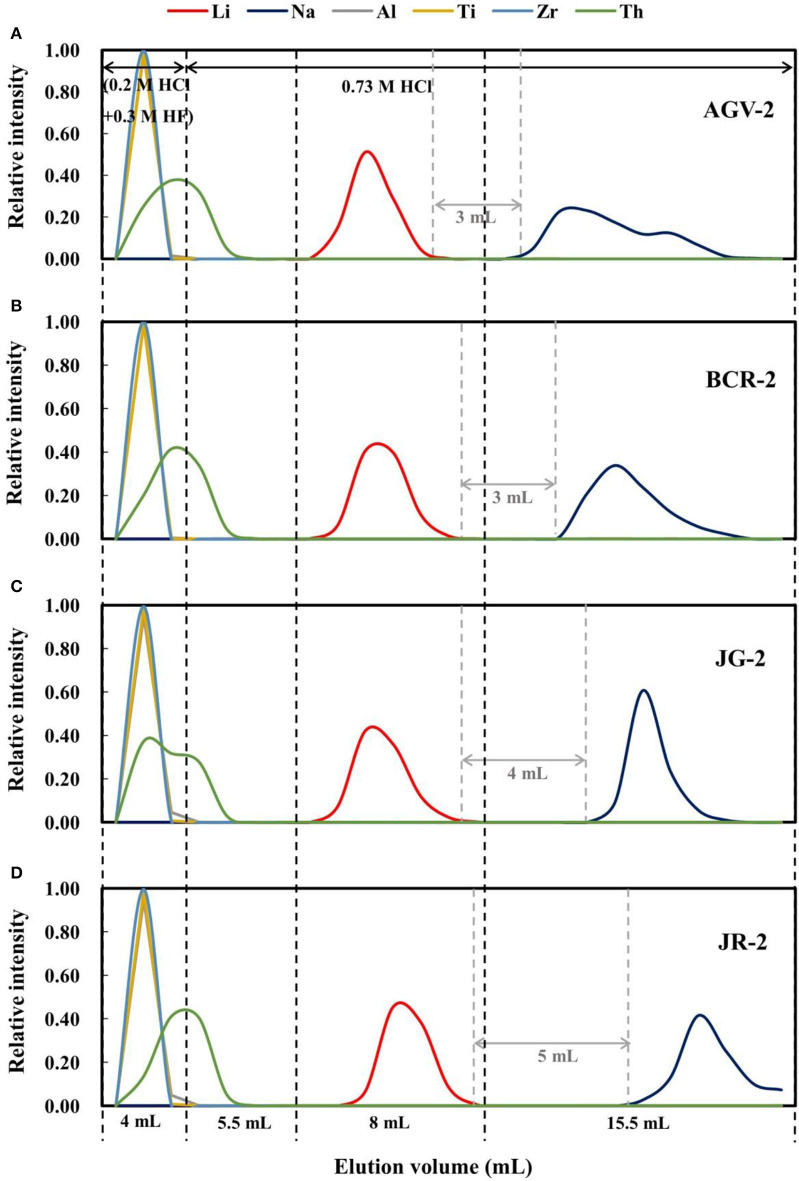
Elution curves for four international rock standard materials on AGMP-50 resin: **(A)** AGV-2; **(B)** BCR-2; **(C)** JG-2; **(D)** JR-2. The relative intensity for all elements represents the percentage content in each elution fraction relative to the total content loaded onto the column.

**Figure 2:**
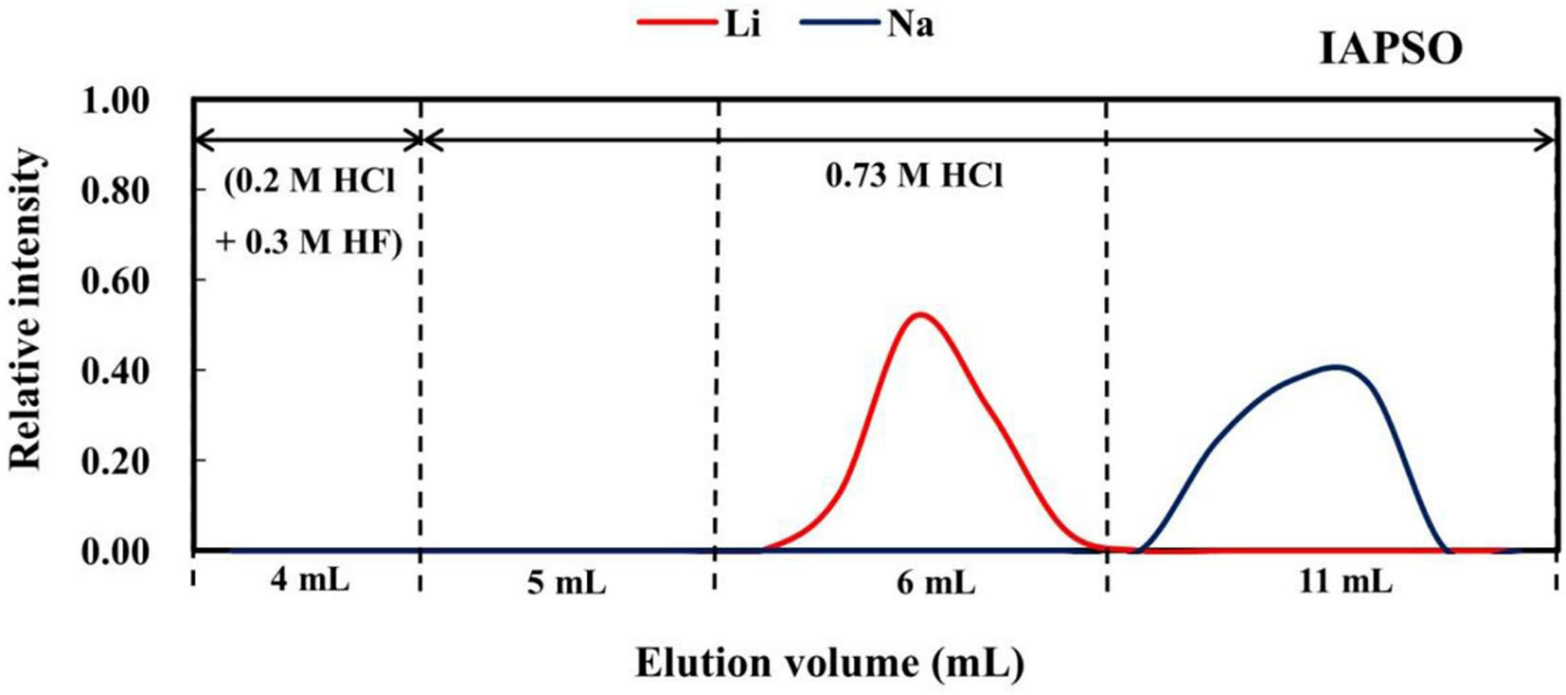
Elution curve for seawater (IAPSO) on AGMP-50 resin.

Approximately 270 ng of Li in each sample (except BHVO-2; 130 ng of Li) was loaded onto the resin in 0.5 mL of 2.5 M HCl, such that the total cation quantities loaded were 0.06–0.6 meq (i.e., <10% of the total resin capacity). Prior to this, the resin was washed with 20 mL of 6 M HCl, 20 mL of 8 M HNO_3_, and 5 mL of Milli-Q water, and then conditioned with 1 mL of 0.2 M HCl. Before collecting Li, 4 mL of 0.2 M HCl + 0.3 M HF was added to remove Al and high-field-strength elements, such as Ti, Zr, and Th. Following this, 5.5 mL of 0.73 M HCl was added, and then Li was collected in 8 mL of 0.73 M HCl for the rock standards (Figure 1). However, for the seawater standard, 5.0 mL of 0.73 M HCl was added, before Li was collected in another 6 mL of 0.73 M HCl (Figure 2).

**Figure 3:**
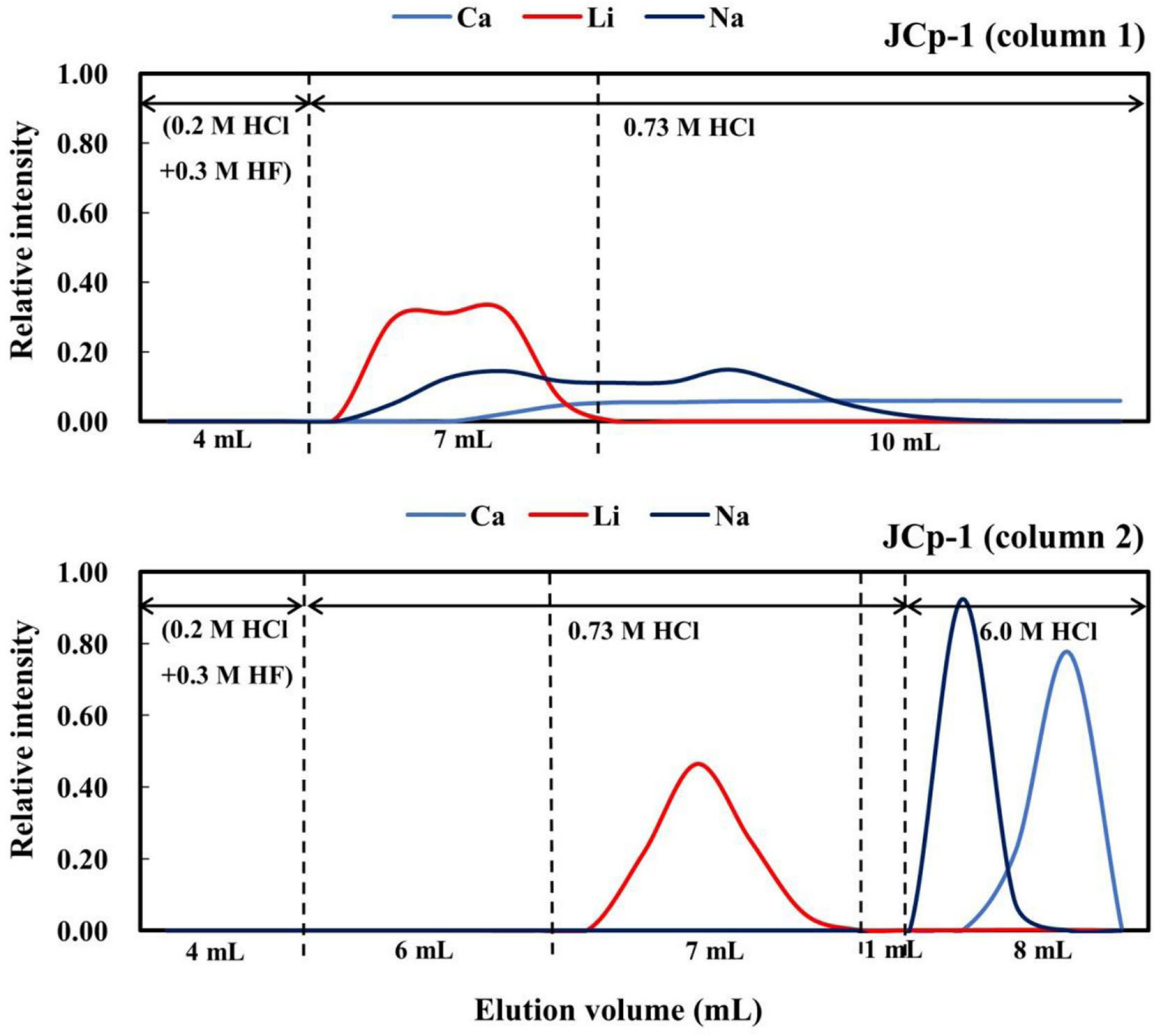
Elution curves for the carbonate reference material JCp-1 on AGMP-50 resin.

Given that the Li content of JCp-1 is very low and its Ca content is high, a two-column separation method was developed for this standard. In this procedure, 4 mL of 0.2 M HCl + 0.3 M HF was first added, and Li was collected in 7 mL of 0.73 M HCl. This resulted in removal of 93% of the Ca and 56% of the Na. The Li cut was then dried and re-dissolved in 0.5 mL of 2.5 M HCl, and loaded onto the same pre-cleaned column for the second separation step. In this step, 4 mL of 0.2 M HCl + 0.3 M HF and 6 mL of 0.73 M HCl were added, and then Li was collected in another 7 mL of 0.73 M HCl (Figure 3).

The detailed Li chemical separation procedures are described in Tables 1, 2. The collected Li was dried on a hotplate at 120°C, and then a few drops of concentrated HNO_3_ were added to the Li cut, which was then evaporated to dryness again. Finally, the Li cuts were re-dissolved in 2% m/m HNO_3_ for Li isotope measurements. The entire Li purification process for the rock and seawater standards takes 0.5 d, and for the carbonate samples takes 1 d. The total chemical procedural blank is <0.1 ng Li. Assuming the isotopic composition of the blank was 30%0 different from the respective sample composition (see published Li isotope compositions of procedural blanks from Jeffcoate et al., 2004 and Rosner et al., 2007), a 0.1 ng Li-blank affecting a 270 ng Li (or 60 ng Li) sample caused a shift of about 0.01%0 (or 0.05%0) in ^7^Li/^6^Li, which is negligible relative to the 60–270 ng of processed Li. ICP-MS analysis showed that the Li recovery is close to 100% (>99%). The chemical procedure for different types of samples is chosen according to the matrices of the samples. For example, for shale or sediment sample, whose matrices are similar to rock samples, the chemical procedure for rocks should be chosen for them. While the separation procedure of carbonate represents the procedure of sample matrix with high Ca and low Li (e.g., [Li] <1 μg g^−1^; [CaO] up to 50%m/m), and the separation procedure of seawater represents the procedure of sample matrix with high Na and low Li (e.g., [Li] <1 μg g^−1^; [Na]/[Li] up to 5 × 10^5^, one order of magnitude higher than the [Na]/[Li] of rock sample).

**Table 1:** Single-column Li separation procedures for rock and seawater samples using AGMP-50 resin.

**Step**	**Details**	**Eluent**	**Volume (rock samples; mL)**	**Volume (seawater; mL)**
Step 1	Resin washing	6 M HCl	20	20
		8 M HNO_3_	20	20
		Milli-Q water	5	5
Step 2	Condition	0.2 M HCl	1	1
Step 3	Loading sample		0.5	0.5
Step 4	Washing	0.2 M HCl + 0.3 M HF	4	4
		0.73 M HCl	5.5	5.0
Step 5	Collecting Li	0.73 M HCl	8	6
Step 6	Washing	6 M HCl	25	25

**Table 2:** Two-column Li separation procedures for carbonate samples using AGMP-50 resin.

**Step**	**Details**	**Eluent**	**Volume (Column 1; mL)**	**Volume(Column 2; mL)**
Step 1	Resin washing	6 M HCl	20	20
		8 M HNO_3_	20	20
		Milli-Q water	5	5
Step 2	Condition	0.2 M HCl	1	1
Step 3	Loading sample		0.5	0.5
Step 4	Washing	0.2 M HCl + 0.3 M HF	4	4
		0.73 M HCl	0	6.0
Step 5	Collecting Li	0.73 M HCl	7	7
Step 6	Washing	6 M HCl	25	25

All digestions and chemical separations were carried out in a class 100 clean hood at the State Key Laboratory of Isotope Geochemistry, Guangzhou Institute of Geochemistry, Chinese Academy of Sciences (GIG-CAS), Guangzhou, China.

### Instrumentation

Lithium isotopic analysis was carried out on a Thermo Fisher Scientific Neptune Plus MC-ICP-MS at GIG-CAS. This instrument is a double-focusing magnetic sector-field mass spectrometer equipped with nine Faraday cups and eight ion counters. All the Faraday collectors are connected to amplifiers with 10^11^ Ω resistors. Sample solutions in 2% m/m HNO_3_ were introduced through a PFA nebulizer (50 μL/min; ESI) and a quartz cyclonic–Scott dual spray chamber. An X skimmer cone was used instead of a H skimmer cone, to improve the instrument sensitivity. The total Li isotope intensity for a 100 ng g^−1^ standard solution of NIST L-SVEC is ~1.75 V on mass ^7^Li. Details of the typical MC-ICP-MS operating parameters are summarized in Table 3.

**Table 3:** Typical MC-ICP-MS operating parameters for Li isotopic measurements.

**Parameter**	**Value**
RF forward power	1015 W (optimized daily)
Cooling gas	16 L/min
Auxiliary gas	1.03 L/min (optimized daily)
Sample gas	1.019 L/min (optimized daily)
Extraction voltage	−2000 V
Focus voltage	−614 V
Accelerating voltage	10 kV
Detection system	Faraday cups
Nebulizer	ESI PFA-50 (~50 μL min^−1^)
X-position	−2.850 mm (optimized daily)
Y-position	−2.590 mm (optimized daily)
Z-position	2.580 mm (optimized daily)
Peri-pump	2 rpm
Instrument resolution	Low resolution
Integration time	4.194 s
Idle time	3 s

### Measurement Strategies

The L4 and H4 Faraday collectors were used to measure ^6^Li and ^7^Li, respectively. The sample–standard–bracketing (SSB) technique was used for mass bias correction and NIST L-SVEC standard solution was selected as the calibrator. Like previous studies (Jeffcoate et al., 2004), substantial instrumental mass bias resulted in a measured ^7^Li/^6^Li for L-SVEC of ~15.5 in this study, as compared with its actual value of 12.02 (Flesch et al., 1973). δ^7^Li was calculated as follows:

δ7Li(‰)=(L7i/L6isample(L7i/L6iL−SVEC1+L7i/L6iL−SVEC2)/2−1)×1000.

where L-SVEC1 and L-SVEC2 are the standard before and after the sample, respectively. All ^7^Li/^6^Li ratios in this equation were blank-corrected to account for instrumental memory effects.

To reduce instrumental mass bias differences between the reference materials and standard solution during Li isotopic measurements, the concentrations were matched at 100 ng·mL^−1^. Data acquisition involved measurement of one block with 50 cycles. The integration time for each cycle was 4.194 s, and the 50 scans took ~4 min.

## Results and Discussion

### Lithium Purification by Different Cation Exchange Resins

**Figure 4:**
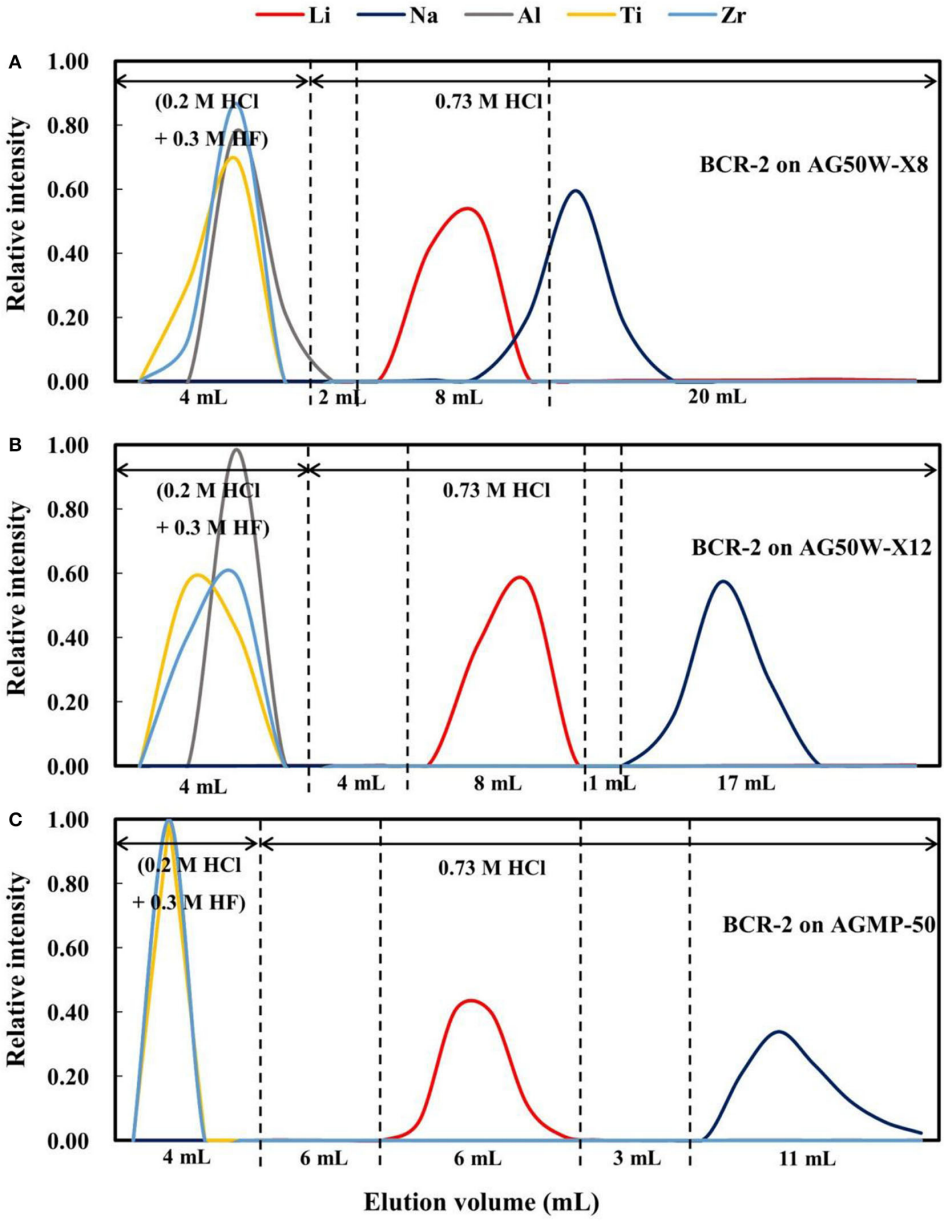
**(A)** The elution curves for BCR-2 on AG50W-X8 resin; **(B)** The elution curves for BCR-2 on AG50W-X12 resin; **(C)** The elution curves for BCR-2 on AGMP-50 resin.

Two types of cation exchange resin, AG50W-X8 (BioRad™; 200–400 mesh) and AG50W-X12 (BioRad; 200–400 mesh) were selected to compare with the AGMP-50 resin (BioRad; 200–400 mesh). The elution curves for BCR-2 on AG50W-X8 resin are shown in Figure 4A, which reveals that the Li elution curve partly overlaps the Na elution curve, making it difficult to completely separate Li from Na. However, Li can be effectively separated from Na on AG50W-X12 resin (Figure 4B), although the elution curves for Li and Na are only narrowly separated (1 mL), as compared with 3 mL for the AGMP-50 resin (Figure 4C). Considering that the elemental elution curves vary slightly with rock type (Figure 1), the AGMP-50 resin provides the best Li and Na separation.

### Li Purification With Different Eluents

**Figure 5:**
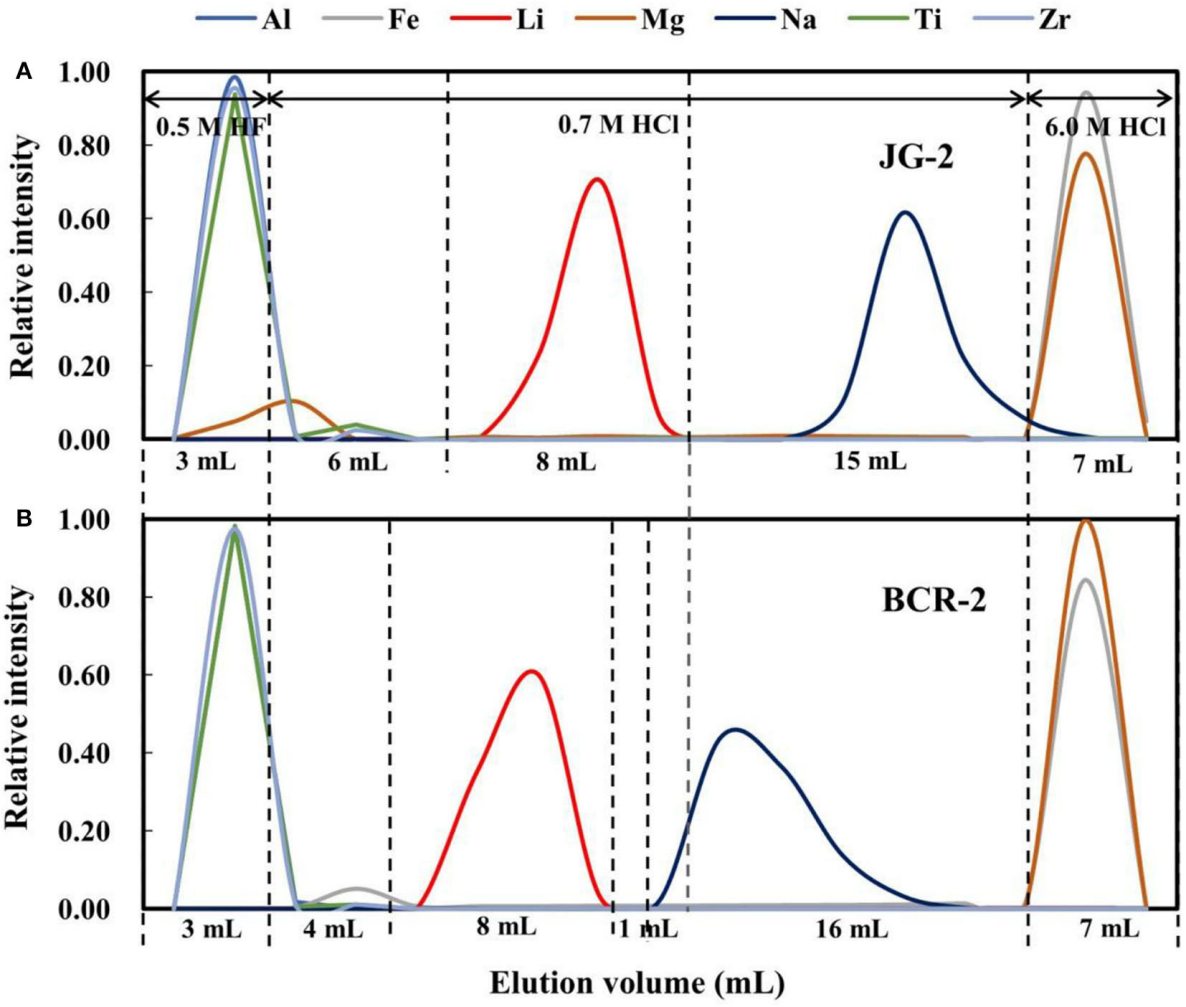
Elution curves for **(A)** JG-2 and **(B)** BCR-2 on AGMP-50 using 0.5 M HF and 0.7 M HCl as an eluent.

In our original experiments, 0.5 M HF was used as the eluent to remove Al and high-field-strength elements, and 0.7 M HCl was used to separate Li from other major matrix elements and, in particular, Na. However, this purification method did not effectively separate Li from Na (Figure 5B). In addition, Li was eluted significantly earlier for basalt than granite (by 2 mL), resulting in Na being present in the Li cut, if the same elution scheme is used for the different rock types (Figure 5). In addition, Mg was eluted with 0.5 M HF (Figure 5A), which may be due to trace MgF_2_ precipitation. This also results in Mg being present in the Li cut.

As such, a mixture of 0.3 M HF + 0.2 M HCl was chosen as the eluent to remove Al and high-field-strength elements, and then 0.73 M HCl was used to separate Li from Na. Figure 1 shows elution curves of Li for different types of rocks, and that Li is eluted earlier for AGV-2 and later for JR-2 in 0.73 M HCl. Moreover, the gaps between the Li and Na elution curves are different, which may be due to variable amounts of matrix in the samples. Given the rock standards have variable Li contents, and we loaded 270 ng of Li in each case, the total ion quantities of each rock sample loaded was different. Given the equivalent weight for common silicate rocks of about 55 mg meq^−1^ (excluding Si) (Gao and Casey, 2012) and, as such, the total ion quantities of AGV-2, BCR-2, JG-2, and JR-2 loaded were 0.45, 0.55, 0.12, and 0.06 meq, respectively. The higher the loaded ion quantity, the narrower the gap between the Li and Na elution curves, which was 3 mL for AGV-2 and BCR-2 (Figure 1). In contrast, where lower ion quantities were loaded on the resin, the gap was 4 mL for JG-2 and 5 mL for JR-2.

In theory, a 5–6 mL elution gap is needed to ensure the Li cut with full recovery for each individual sample. However, given that the different types of rocks have different elution curves, an 8 mL gap is more suitable. A gap of at least 3 mL was achieved between the Li and Na elution curves in our study.

### Effect of Lithium Load on Purification

**Figure 6:**
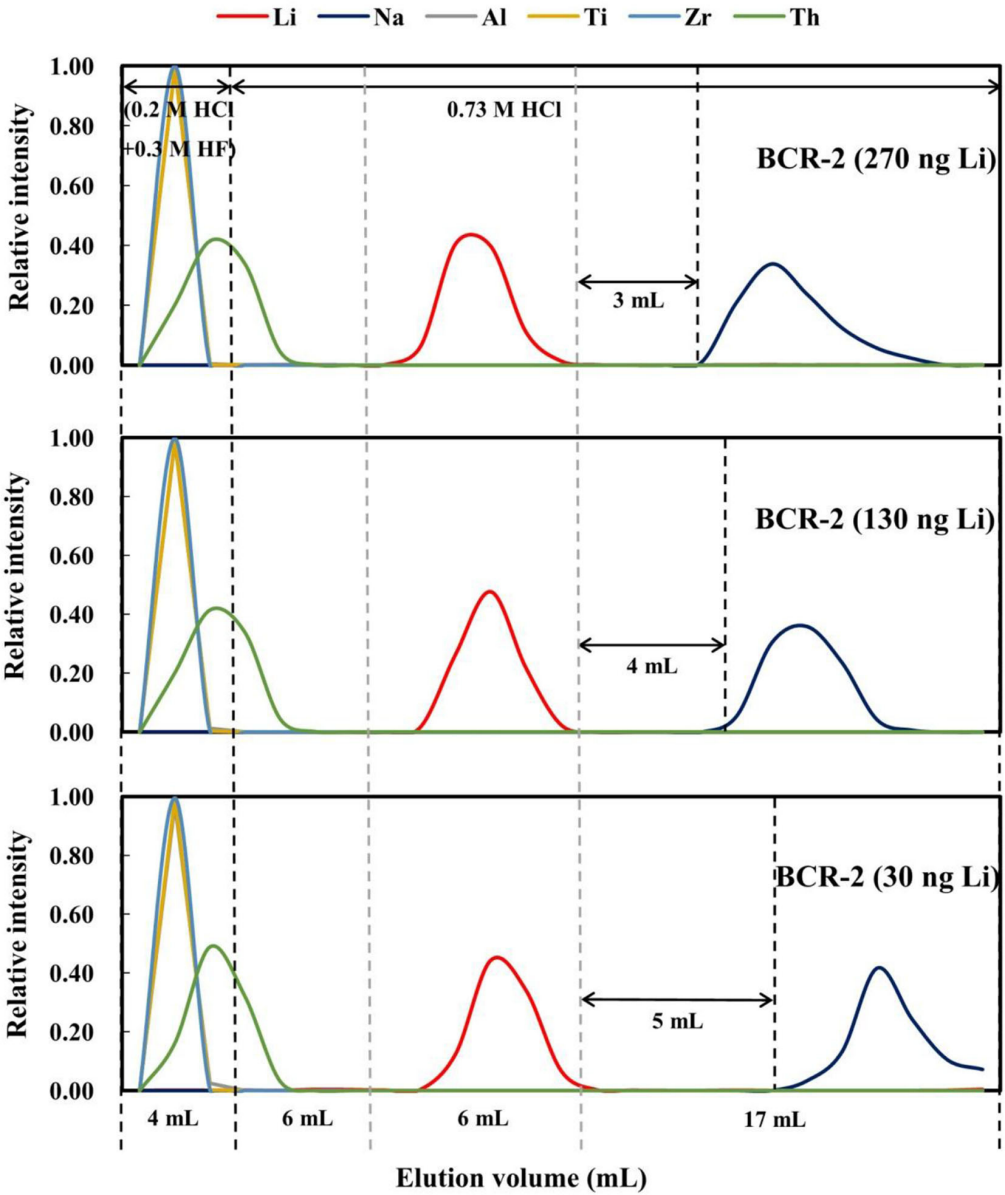
Elution curves for BCR-2 with different Li loads (270, 130, and 30 ng Li) on AGMP-50 resin.

BCR-2 containing 270, 130, and 30 ng of Li were used to evaluate the effect of different Li loading amounts on Li purification. Lithium was preferentially eluted out in the sample containing 270 ng Li and last leached out in the sample containing 30 ng of Li (Figure 6). The gaps between the Li and Na elution curves were also different, being 3 mL for 270 ng of Li, 4 mL for 130 ng of Li, and 5 mL for 30 ng of Li. These results are consistent with those obtained on the different types of rock standards.

### Effect of Loading Volume on Lithium Purification

**Figure 7:**
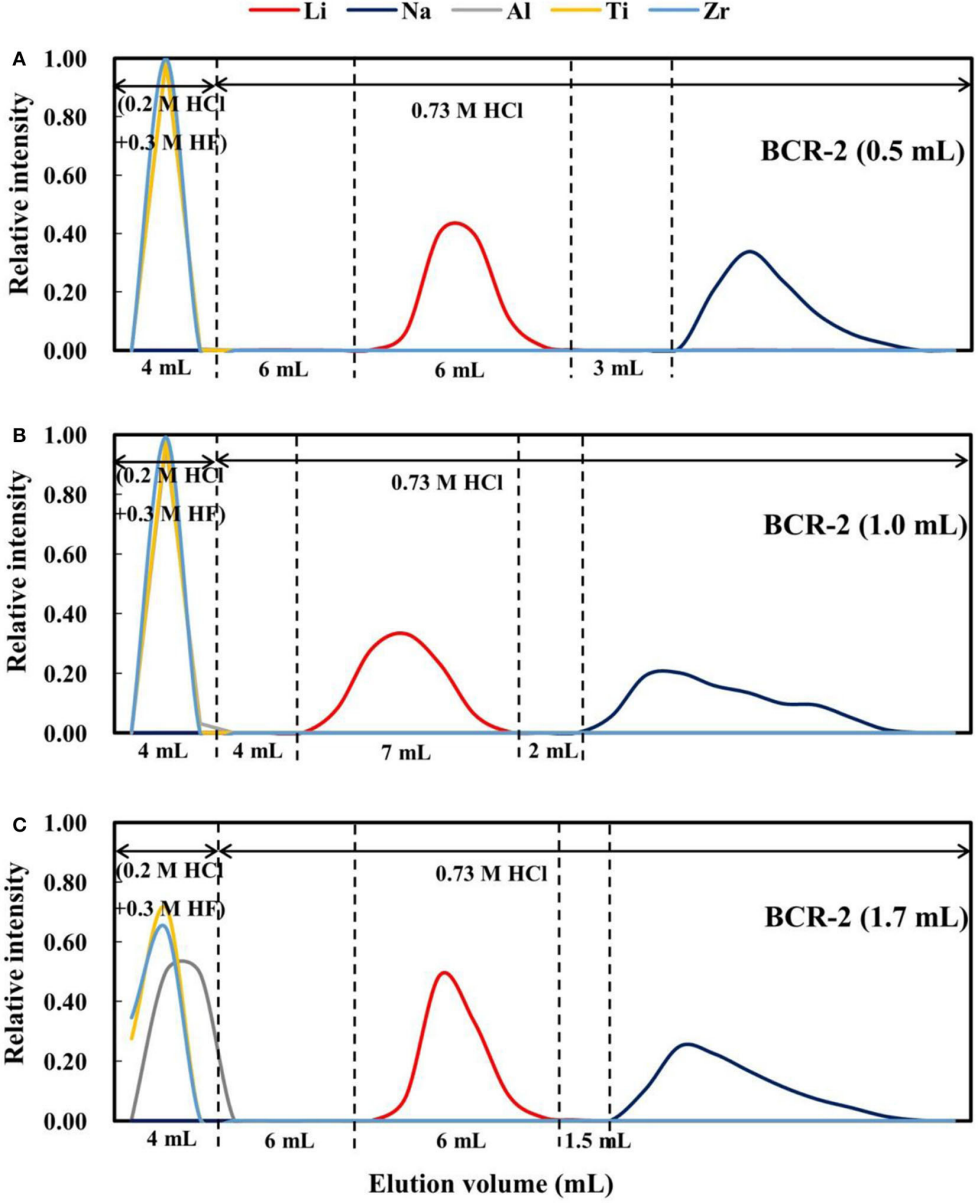
Elution curves for BCR-2 with different loading volumes on AGMP-50 resin. **(A)** 270 ng Li in 0.5 mL of 2.5 M HCl; **(B)** 270 ng Li in 1.0 mL of 2.5 M HCl; **(C)** 270 ng Li in 1.7 mL of 0.73 M HCl.

It has been documented that the loading volume has a considerable effect on elemental behavior during ion chromatography, such as the retention volume and peak width. For example, (Ren et al., 2013) reported that with an increase in injection volume, the retention time or volume and peak width increase. To evaluate this effect on Li purification with AGMP-50 resin, 270 ng of Li dissolved in 0.5 and 1.0 mL of 2.5 M HCl was processed through our procedure. The elution curves (Figure 7) indicate that when the load volume increases from 0.5 to 1.0 mL, the peak width and separation of the Li and Na elution curves become wider and smaller (3–2 mL), respectively. However, such differences might also result from the different acidity of the loads in 0.5 and 1 mL of 2.5 M HCl. As such, an additional 270 ng of Li in 1.7 mL of 0.73 M HCl, with an acidity equivalent to 270 ng of Li in 0.5 mL of 2.5 M HCl, was loaded. When the loading volume increases to 1.7 mL, the peak widths of Al, high-field-strength elements, and Na are clearly wider, but the peak width of Li changes little (Figure 7C). In addition, the gap between the Li and Na elution curves decreased to 1.5 mL. Therefore, increasing the loading volume does not enhance the separation of Li from Na.

### Instrument Stability

**Figure 8:**
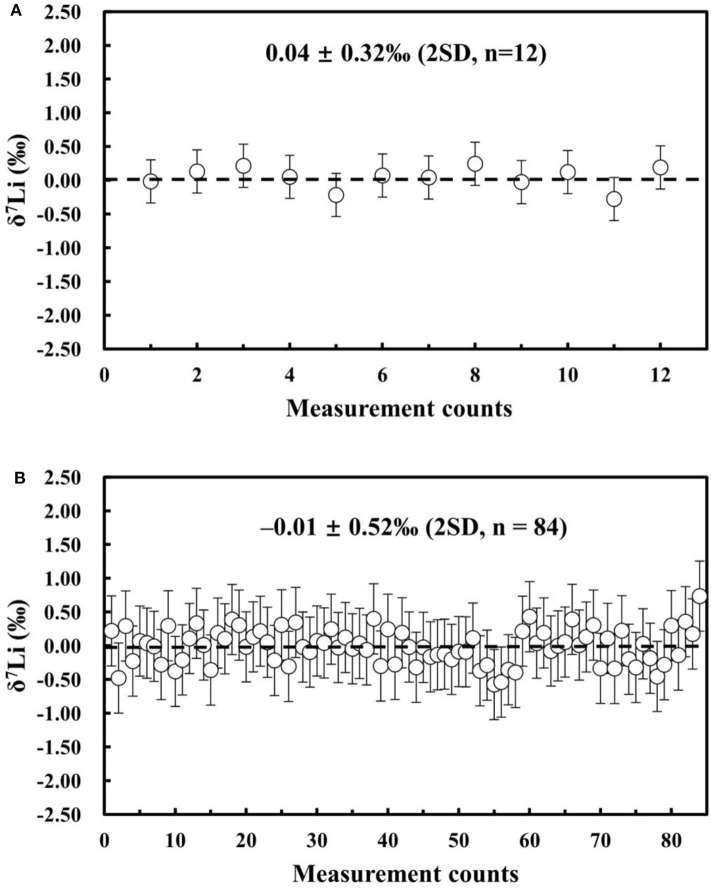
**(A)** Instrument stability of ~10 h (δ^7^Li of NIST L-SVEC) and **(B)** long-term external reproducibility for δ^7^Li (NIST L-SVEC) in the past 12 months.

We evaluated the stability of the MC-ICP-MS instrument by repeatedly measuring the Li isotopic composition of the NIST L-SVEC standard solution. During this test, we maintained a constant washout time and matched the Li concentration and acidity of the analyzed NIST L-SVEC standard solutions. Analytical uncertainties are reported as 2SD (standard deviation of n repeated sample analyses) and 95% confidence interval (c.i.). The 95% c.i. is calculated from the standard deviation of *n* repeated sample analyses (*n* < 20) and is corrected by the Student's t factor ($95\%\;c.i.\;=t\frac{SD}{\sqrt{n}}$). The short-term external reproducibility of ~10 h was δ^7^Li = 0.04 ± 0.32%0 (2SD; *n* = 12), and the long-term external reproducibility was δ^7^Li = −0.01 ± 0.52%0 (2SD; *n* = 84) over 1 year (Figure 8). Therefore, high-precision Li isotope measurements can be obtained using our Neptune Plus MC-ICP-MS.

### Effects of Intensity and Acidity on Lithium Isotope Measurements

**Figure 9:**
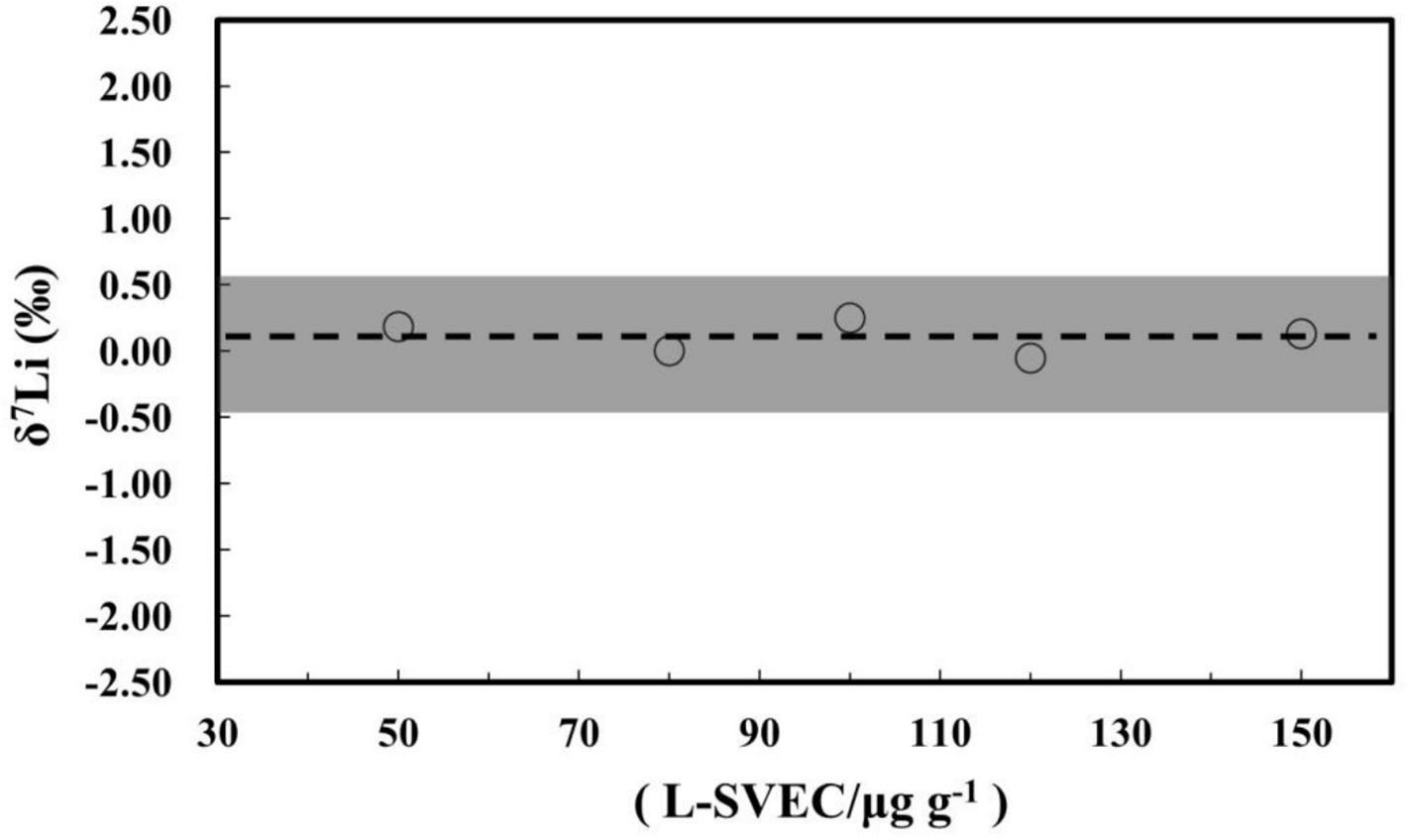
Intensity effect on Li isotope measurements of L-SVEC.

A concentration-dependent effect on Li isotopic measurements by MC-ICP-MS has previously been documented (Bryant et al., 2003; Magna et al., 2004; Rosner et al., 2007; Lin et al., 2015; Hu and Teng, 2019). As such, five different concentrations of the NIST L-SVEC standard solution were analyzed (50, 80, 100, 120, and 150 ng g^−1^). The Li isotope ratio was measured relative to a NIST L-SVEC standard solution with 100 ng g^−1^ Li in 2% m/m HNO_3_. Our NIST L-SVEC solutions with Li concentrations of 50–150 ng g^−1^ have the same Li isotope compositions, with an average δ^7^Li of 0.10 ± 0.25%0 (2SD). Therefore, within this Li concentration range, high-precision measurement of Li isotopes can be obtained (Figure 9).

**Figure 10:**
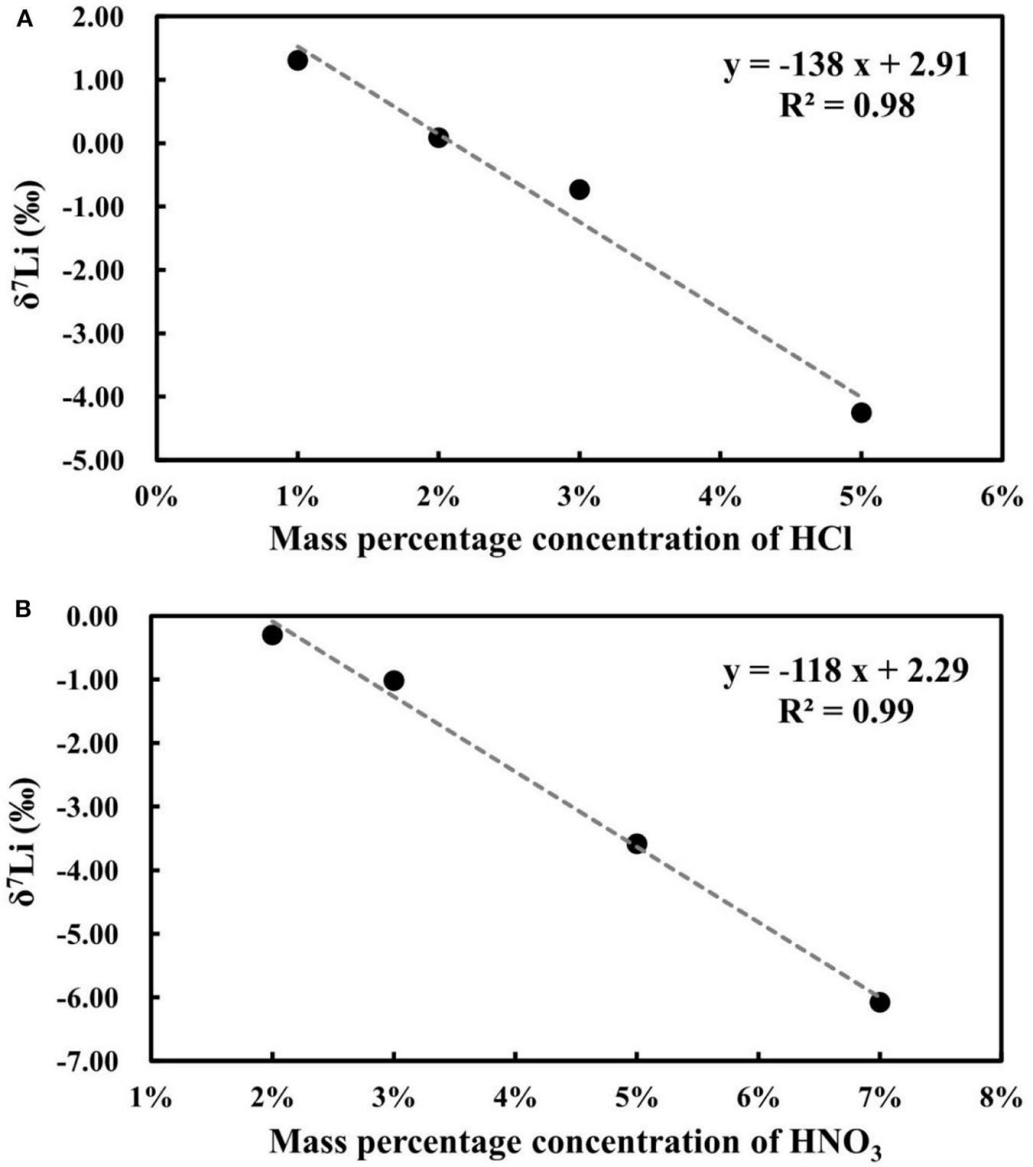
**(A)** The effects of HCl matrix on Li isotope measurement; **(B)** The effects of HNO3 matrix on Li isotope measurement.

A previous study documented that different concentrations of acid can cause matrix effects and changes in mass bias during Li isotope measurements (Bryant et al., 2003; Lin et al., 2015; Hu and Teng, 2019). The acidity effect was evaluated by analyzing 100 ng g^−1^ of NIST L-SVEC standard solution in different concentrations of HNO_3_ and HCl, ranging from 2 to 7% m/m for HNO_3_ and 1 to 5% m/m for HCl. All the results were obtained relative to 100 ng g^−1^ of NIST L-SVEC Li standard solution in 2% m/m HNO_3_. A negative linear correlation with *R*^2^ = 0.99 is evident between the δ^7^Li values of the NIST L-SVEC in 2% m/m HNO_3_ and different concentrations of HNO_3_, and a similar correlation with *R*^2^ = 0.98 was observed between the δ^7^Li values of the NIST L-SVEC in 2% m/m HCl and different concentrations of HCl (Figure 10). Based on these results, it is clear that significant Li isotope inaccuracies can result if the samples and standard are not in the same acid type and concentration, which therefore requires the acidity of the sample to be well-matched with that of the standard solution.

### Matrix Effects on Lithium Isotope Measurements

**Figure 11:**
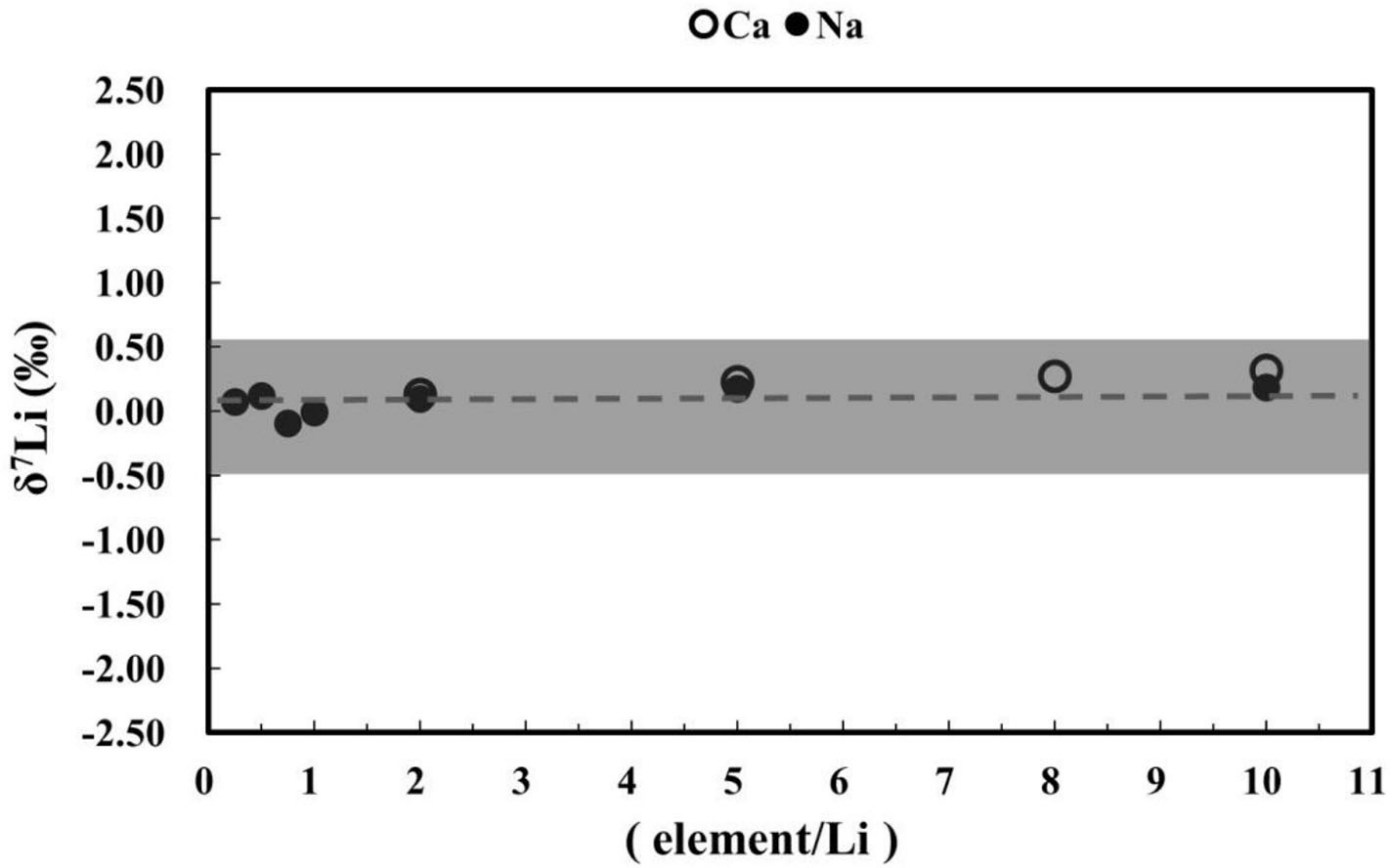
Effects of Na and Ca on Li isotope measurements.

Sodium and calcium can compromise high-precision Li isotope measurements by MC-ICP-MS. For example, Li W. et al. (2018) and Hu and Teng (2019) reported that accurate measurements of Li isotopes on a Neptune Plus or Nu MC-ICP-MS require Na/Li < 1 and Ca/Li < 0.5. Rosner et al. (2007) suggested that Na/Li < 5 can also yield accurate data, whereas Lin et al. (2019) reported that Na/Li and Ca/Li ratios can be as high as 10 and still yield accurate Li isotope data. We assessed the matrix effects of Na and Ca on Li isotope measurements using an elemental doping method. In this test, 100 ng g^−1^ L-SVEC Li solutions were doped with known amounts (25, 50, 75, 100, 200, 500, and 1,000 ng g^−1^) of Na and (200, 500, 800, and 1,000 ng g^−1^) of Ca, with Na/Li = 0.25–10 and Ca/Li = 2–10. The test showed no detectable matrix effects from Na and Ca on Li isotope measurements, even at Na/Li and Ca/Li = 10 (Figure 11), which is consistent with the results of Lin et al. (2019). Given that the Na/Li and Ca/Li ratios were < 1 in our analyzed rock and seawater samples, and Ca/Li was < 2 for JCp-1, our data are not compromised by matrix effects.

### Lithium Isotopic Compositions of Geological Standard Materials

**Figure 12:**
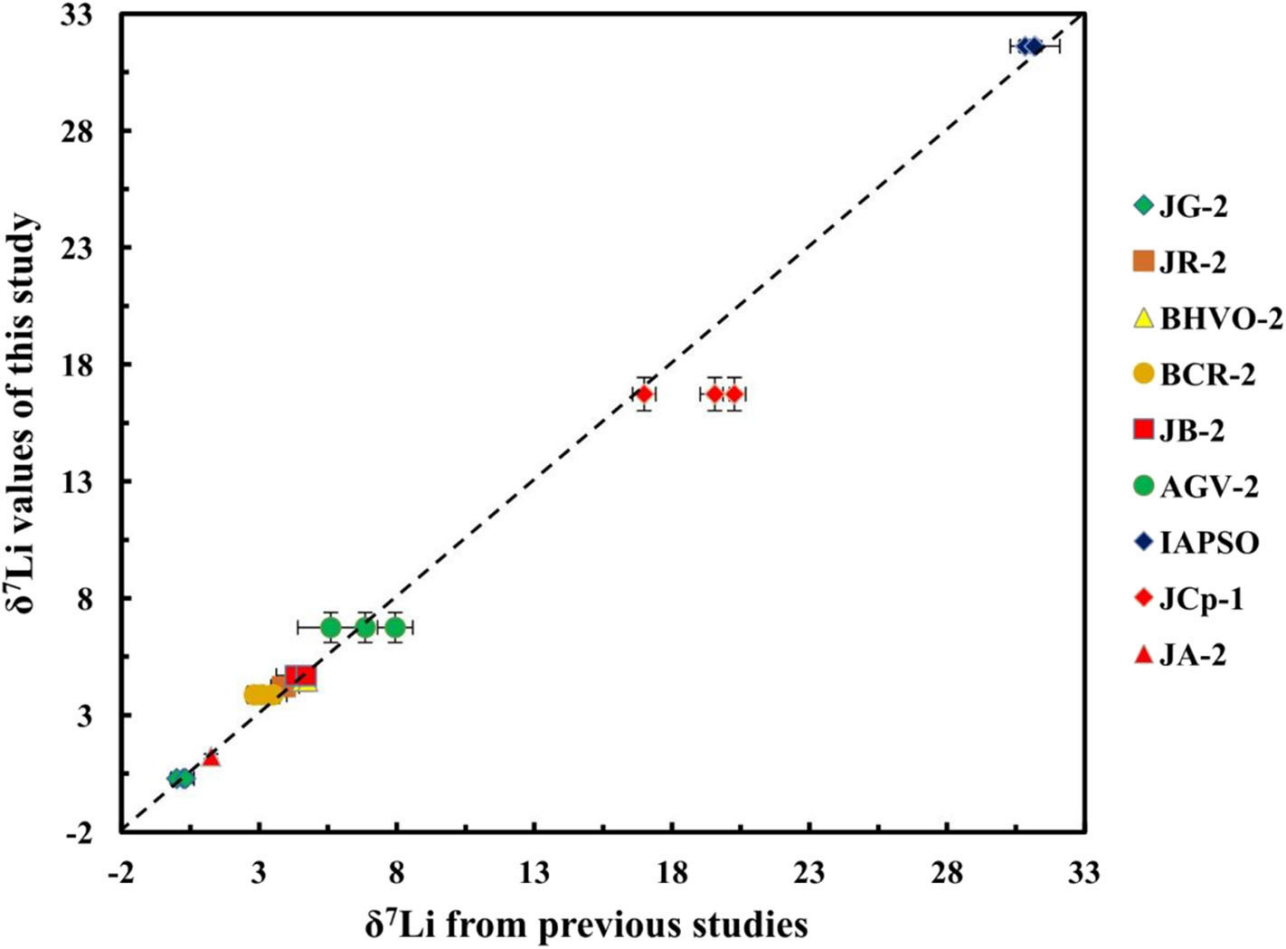
Lithium isotope compositions of geological standard materials.

All the geological reference materials were analyzed in duplicate (column chemistry) and in quadruplicate (instrumental measurements). The average δ^7^Li values obtained were as follows: JG-2 = 0.30 ± 0.20%0 (2SD; *n* = 7), JR-2 = 4.23 ± 0.34%0 (2SD; *n* = 5), JB-2 = 4.70 ± 0.15%0 (2SD; *n* = 4), BHVO-2 = 4.46 ± 0.42%0 (2SD; *n* = 5), BCR-2 = 3.88 ± 0.36%0 (2SD; *n* = 4), AGV-2 = 6.76 ± 0.64%0 (2SD; *n* = 6), and IAPSO = 31.62 ± 0.22%0 (2SD; *n* = 4) (Table 4). These data are in excellent agreement with previously published data for these standards (Figure 12) (Jeffcoate et al., 2004; Magna et al., 2004; Rosner et al., 2007; Pogge von Strandmann et al., 2008; Genske et al., 2014; Ryu et al., 2014; Tang et al., 2014; Lin et al., 2015, 2019; Phan et al., 2016; Rodovská et al., 2016; Bohlin et al., 2017; Bastian et al., 2018; Liu et al., 2018; Li W. et al., 2018). The carbonate reference material JCp-1 yields δ^7^Li values of 16.74 ± 0.71%0 (2SD; *n* = 5), which are consistent with the published data (16.99 ± 0.42%0, *n* = 6) (Lin et al., 2019) and lower than the reported data (19.56–20.27%0) (Bohlin et al., 2017; Li W. et al., 2018). Based on the above reported data, our study supports that the Li isotopes of JCp-1 are heterogeneous, as suggested by Bastian et al. (2018) and Lin et al. (2019), which suggests that carbonate reference material of JCp-1 should be treated with caution for inter-laboratory comparisons of Li isotope composition. The new recommended δ^7^Li value of JA-2 is 1.25 ± 0.10%0 (2SD; *n* = 4), which are presented as reference values for neutral rock reference material and inter-laboratory calibration for future research.

**Table 4:** Li isotopic compositions of standard reference materials.

**Sample ID**	**Sample type**	**δ^7^Li (%0)**	**2SD (%0)**	**95% c.i. (%0)**	**n**
JG-2	Granite	0.30	0.20	0.12	5
JR-2	Rhyolite	4.23	0.34	0.21	5
BHVO-2	Basalt	4.46	0.42	0.26	5
BCR-2	Basalt	3.88	0.36	0.29	4
JB-2	Basalt	4.70	0.15	0.12	4
JA-2	Andesite	1.25	0.10	0.08	4
AGV-2	Andesite	6.76	0.64	0.33	6
IAPSO	Seawater	31.62	0.22	0.18	4
JCp-1	Aragonite	16.74	0.71	0.44	5

*n corresponds to “number of measurements”*.

## Conclusions

A simple and rapid method has been developed to purify Li from other matrix elements using AGMP-50 cation exchange resin, followed by high-precision Li isotope measurements by MC-ICP-MS. Compared with previously reported Li purification methods, our procedure purifies large Li amount but uses smaller reagent volumes and is simple. Lithium isotope compositions reported for a suite of geological reference materials are consistent with previously published data, which demonstrates the veracity of our techniques. Therefore, this method is ideally suited for Li separation from multiple types of geological samples for isotopic analysis.

## Data Availability Statement

The raw data supporting the conclusions of this article will be made available by the authors, without undue reservation.

## Author Contributions

All authors listed have made a substantial, direct and intellectual contribution to the work, and approved it for publication.

## Conflict of Interest

The authors declare that the research was conducted in the absence of any commercial or financial relationships that could be construed as a potential conflict of interest. The handling editor is currently co-organizing a Research Topic with one of the authors GW, and confirms the absence of any other collaboration.
